# A Novel Effector, FSE1, Regulates the Pathogenicity of *Fusarium oxysporum* f. sp. *cubense* Tropical Race 4 to Banana by Targeting the MYB Transcription Factor MaEFM-Like

**DOI:** 10.3390/jof9040472

**Published:** 2023-04-14

**Authors:** Yongbao Yang, Bang An, Yunfeng Guo, Hongli Luo, Chaozu He, Qiannan Wang

**Affiliations:** 1 Sanya Nanfan Research Institute of Hainan University, College of Tropical Crops, Hainan University, Haikou 570228, China; 2 Hainan Yazhou Bay Seed Laboratory, Sanya 572025, China

**Keywords:** *Fusarium oxysporum*, banana wilt disease, effector, interaction, hypersensitive reaction

## Abstract

Phytopathogenic fungi secretes a range of effectors to manipulate plant defenses. *Fusarium oxysporum* f. sp. *cubense* tropical race 4 (Foc TR4) is a soil-borne pathogen that causes destructive banana wilt disease. Understanding the molecular mechanisms behind Foc TR4 effectors and their regulation of pathogenicity is helpful for developing disease control strategies. In the present study, we identified a novel effector, *Fusarium* special effector 1 (FSE1), in Foc TR4. We constructed *FSE1* knock-out and overexpression mutants and investigated the functions of this effector. In vitro assays revealed that FSE1 was not required for vegetative growth and conidiation of Foc TR4. However, inoculation analysis of banana plantlets demonstrated that knock-out of *FSE1* increased the disease index, while overexpression of *FSE1* decreased it. Microscope analysis suggested that FSE1 was distributed in the cytoplasm and nuclei of plant cells. Furthermore, we identified an MYB transcription factor, MaEFM-like, as the target of FSE1, and the two proteins physically interacted in the nuclei of plant cells. In addition, Transient expression of MaEFM-like induced cell death in tobacco leaves. Our findings suggest that FSE1 is involved in the pathogenicity of Foc TR4 by targeting MaEFM-like.

## 1. Introduction

*Fusarium oxysporum* Schlecht. is a soil-borne pathogen that is widely distributed around the world and infects a wide range of plants, resulting significant losses in crops such as tomato, cotton, and banana [1,2]. The pathogen infects and colonizes the vascular systems of its hosts, causing vascular browning, progressive wilting, defoliation, and plant death [3]. The *F. oxysporum* species are categorized into various formae speciales (f. sp.), with individual isolates causing disease only on one or a few plant species [4]. *F. oxysporum* f. sp. *cubense* (Foc) races are responsible for banana (*Musa* spp.) wilt disease, also named ‘Panama disease’. Several races of Foc have been recognized to date, among which Foc tropical race 4 (Foc TR4) can infect the primary commercial banana cultivar, Cavendish, leading to significant economic loss in banana plantations worldwide [5]. Given that Foc TR4 is soil-borne and possesses strong stress resistance, there are still no effective management strategies against the banana wilt disease [6].

Pathogenic microorganisms have evolved sophisticated strategies to evade, overcome, or manipulate host immunity systems during long periods of co-evolution with plants. One such strategy is to secrete small proteins known as effectors. Effectors have been identified in bacteria, oomycetes, and fungi [7,8], with bacterial effectors being conserved in sequences and delivered into host cells via specialized secretion systems such as type III [9]. Oomycete pathogens secrete effectors with consensus N-terminal sequence motifs such as RXLR, LFLAK, and CHXC amino acid sequences via haustoria [10]. Whereas fungal effectors are variable in motifs and domains, and are secreted via multiple systems [10], making them diverse and difficult to be predicted.

Via the *F. oxysporum* f. sp. *lycopersici* (Fol)-tomato interaction system, a group of cysteine-rich effectors named secreted in xylem (SIX) were discovered in the xylem sap proteome of tomato plantlets [11,12]. These SIX proteins display low homology with other known proteins and have been found to function as elicitors and/or suppressors of R gene-based plant immunity [13,14,15]. In our previous work, the effector SIX8 was found to be required for the pathogenicity of Foc TR4 to banana [16], and two conserved fungal effectors cerato-platanin 1 (CP1) could directly interact with banana pathogenesis-related protein 1 (PR1), contributing to FocTR4 pathogenicity [17]. Based on secretome analysis, we also identified a series of effector candidates in Foc TR4 [18]. However, the identification of novel effectors and understanding of effectors in the regulation of Foc TR4-banana interaction are still inadequate.

In this study, a novel effector specific to *Fusarium* species was discovered in Foc TR4, which was found to be involved in Foc TR4 pathogenicity and could directly interact with a banana MYB family transcription factor. These findings provided some clues for understanding the pathogenicity of Foc TR4 and the gene-for-gene system of plant immunity.

## 2. Materials and Methods

### 2.1. Bioinformatics Analysis

An effector candidate FSE1 was predicted in Foc TR4 through comparison of the genomes of Foc TR4 and Foc Race 1. The homologous protein sequences of FSE1 were retrieved from the NCBI GenBank database through BLASTP search. The maximum-likelihood phylogenetic tree of FSE1 with the orthologs was constructed with 1000 bootstrap replicates using MEGA 11 [19]. Conserved domains of FSE1 were searched in SMART and Pfam database. Signal peptides were predicted with SignalP 5.0 [20].

### 2.2. Fungal Strains and Culture Conditions

Wild type Foc TR4 strain was maintained on potato dextrose agar (PDA) medium at 28 °C. For the colony growth and conidiation assays, Foc TR4 strains were grown on/in the complete or minimal medium according to our previous work [21].

### 2.3. Vector Construction and Protoplast Transformation

**Figure 1:**
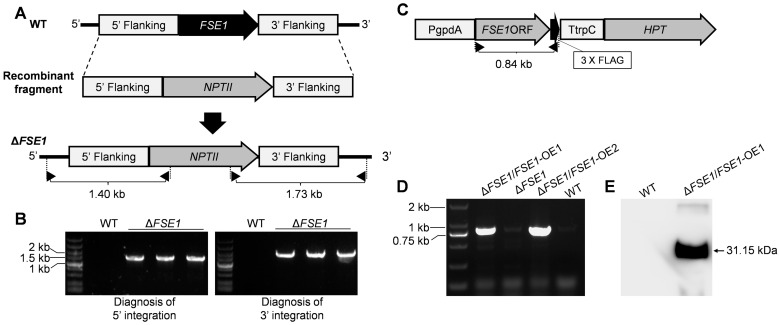
Strategies for construction of the *FSE1* knockout and overexpression mutants. (**A**) The strategies for *FSE1* knock-out. Diagnostic primers for integrations of the recombinant fragments are marked with black triangles. (**B**) Diagnosis for integrations of the recombinant fragments into the *FSE1* locus. (**C**) The expression cassette of *FSE1*. Diagnostic primers for *FSE1-FLAG* nucleotide are marked with black triangles. (**D**) Diagnosis for *FSE1-FLAG* nucleotide. (**E**) Western-blot analysis for expression of FSE1-FLAG protein.

The nucleotide of *FSE1* was knocked out via the homologous recombination strategy as shown in Figure 1. Vector pBS-NEO containing the Neomycin phosphotransferase gene (*NPTII*) was used as a backbone to construct replacement vectors. The up- and down-flanking regions of *FSE1* were ligated with *NPTII* to construct the recombinant fragment (Figure 1A). Then the linearized recombinant fragment was transformed into protoplasts of the WT strain according to the procedures [16]. The transformants resistant to 100 μg mL^−1^ G418 (Sigma-Aldrich, St. Louis, MO, USA) were selected for mutant diagnosis. A two-round PCR diagnosis was conducted to confirm the correct integration of the recombinant fragments into the target locus, using primer pairs with one primer being located out of the flanking fragment, and the other in the selection marker gene (Figure 1A). Homokaryotic mutants were obtained through single conidia isolation.

For the construction of the *FSE1* overexpression (OE) mutant, the open reading frame (ORF) of *FSE1* was ligated into the plasmid pMD-PgTt [21] to construct the expression cassette driven by the promoter of glyceraldehyde-3-phosphate dehydrogenase (gpdA) and the terminator of trpC from *Aspergillus nidulans*. Then the linearized vector was transformed into protoplasts of the FSE1 knock-out mutant strain, and the transformants resistant to 300 μg mL^−1^ Hygromycin B (Sigma-Aldrich, St. Louis, MO, USA) and 100 μg mL^−1^ G418 were selected for PCR diagnosis of FSE1 ORF.

For the construction of the GFP or FSE1-GFP fusion expression mutant, the ORF of GFP was ligated into pMD-PgTt or pMD-PgTt-FSE1 plasmids, respectively. After that, the linearized vector was transformed into protoplasts of WT strain. And the transformants was identified by diagnosis of GFP or FSE1-GFP sequences. All the primers used were listed in Table S1.

### 2.4. Inoculation of Banana Plantlets and Pathogenicity Assay

Pathogenicity assay was carried out as described previously with some modifications [21]. Briefly, Foc TR4 strains were incubated in a liquid complete medium for 3 d, then conidia were collected, washed, and resuspended with ddH_2_O to a final concentration of 10^5^ conidia mL^−1^. Banana plantlets (*Musa acuminata* L. AAA group, ‘Brazilian’) obtained from the Tissue Culture Center of Chinese Academy of Tropical Agricultural Sciences were cultured in the glasshouse. Each banana plantlet was irrigated with 50 mL of conidia suspension for the inoculation. After the inoculation for 5 weeks, the disease symptoms of banana pseudostem were recorded and the disease scores were calculated as described in our previous work [17,21]. Each treatment contained a total of 20 banana plantlets. The plantlets inoculated with ddH_2_O were used as control check (CK). The disease scores were defined as follows: 0 (no symptoms), 1 (some brown spots in the inner rhizome), 2 (less than 25% of the inner rhizome showed browning), 3 (up to 3/4 of the inner rhizome showed browning), and 4 (entire inner rhizome were dark brown). Differences in the distributions of disease scores between treatments were tested for statistical significance by Mann-Whitney tests. The experiment was conducted twice.

### 2.5. RT-qPCR

For the in vitro sample, Foc TR4 were grown in the complete medium for 2 d, and the mycelium were collected for RNA extraction. For the *in planta* samples, the banana plantlets after inoculation with Foc TR4 WT strain for 3, 5 and 7 d were sampled. At each time point, 5 banana plantlets were picked randomly as an independent sample. The root was washed clean, cut from the plants and used for RNA extraction. Total RNA was extracted with RNAprep Pure Plant Plus Kit (TIANGEN Biotech, Beijing, China). First strand cDNA was synthesized with FastKing gDNA Dispelling RT SuperMix (TIANGEN Biotech, Beijing, China). RT-qPCR analysis was performed with the QuantStudio 6 (Thermo Fisher). The relative transcription levels were estimated using the 2^−ΔΔCt^ method with actin coding gene as the endogenous control. Each reaction contained three biological replicates. The primers used are listed in Table S1.

### 2.6. Yeast Two-Hybrid System

The ORF of FSE1 without signal peptide (SP) coding sequence was introduced into pGBKT7 as bait. Then the Matchmaker Gold Yeast Two-Hybrid (Y2H) System (Clontech, Palo Alto, CA, USA) was used to screen the cDNA libraries from the banana root [17] for proteins that interact with FSE1. The Yeast Nitrogen Base without amino acids, the Double dropout supplement -Leu-Trp, and quadruple dropout supplement -Trp-Leu-His-Ade were used for the experiment. To verify the protein interactions, the ORFs of FSE1 and the identified prey proteins were introduced into pGBKT7 and pGADT7 respectively, and both the bait and prey plasmids were co-transformed into yeast strain Y2H Gold. Then the transformed yeast cells were assayed for growth on synthetic dropout (SD)/-Trp-Leu plates and SD/-Trp-Leu-His-Ade plates containing 125 ng mL^−1^ aureobasidin A (ABA). The Yeast Nitrogen Base without Amino Acids was used for the analysis.

### 2.7. Subcellular Localization and Bimolecular Fluorescence Complementation (BiFC)

For investigation of the subcellular localization of FSE1 in the plant cell, its coding sequence was introduced into plasmid pEGAD; For BiFC assay, its coding sequence was introduced into pNC-BiFC-Enn, and the ORF of *MaEFM*-like was introduced into pNC-BiFC-Enc. Then all these plasmids were transformed into the *Agrobacterium tumefaciens* GV3101 respectively, and the *Agrobacterium* harboring plasmids were infiltrated into *Nicotiana benthamiana* leaves for transient expression. After infiltration for two days, the *N. benthamiana* leaves were stained with 4′,6-diamidino-2-phenylindole (DAPI, 5 μg/mL) and were then sampled and observed under the confocal laser scanning microscope (Leica TCS SP8), with excitation of 488 nm argon laser, and emission wavelength range of 505–535 nm.

## 3. Results

### 3.1. FSE1 Is a Special Candidate Effector Conserved in in Fusarium spp.

Based on the Foc TR4 genome, the gene FOIG_03990 was identified and predicted to encode an extracellular secretory protein. The nucleotide sequence of the gene was 882 bp, containing a 771 bp open reading frame separated by two introns, which encodes a protein of 256 amino acids. The recently released genome data generated by the PacBio Sequel platform (accession: GCA_027920445.1) revealed that the gene is located on chromosome 9 of Foc TR4. The predicted protein shows a typical effector characteristic with 16 cysteine residues and a signal peptide (1–19 aa) at its N-terminal (Figure S1). The BLASTP analysis against the NCBI database was conducted to identify homologs proteins in other fungi. The results revealed that the predicted protein is only conserved in in *Fusarium* species but not in other fungi, furthermore, the coding sequence of the protein is also lacing in Foc Race 1, which severely affects most of the banana varieties except Cavendish banana (AAA). The phylogenetic tree analysis also confirmed the conservation of the protein in *Fusarium* species (Figure S2). Moreover, the protein did not contain any identified domains as revealed by searching in Pfam. Furthermore, the expression profile of *FSE1* showed that the gene was nearly not expressed in the in vitro cultured mycelium, but it was significantly up-regulated after colonization of the banana root, suggesting its importance in the pathogenicity of Foc TR4 (Figure S3). These data indicated that the protein is a special candidate effector conserved in in *Fusarium* species and might play important roles in the pathogenicity of Foc TR4 to Cavendish banana, therefore, the protein was named FSE1 (*Fusarium* special effector 1).

### 3.2. Construction of FSE1 Knock-Out and Over-Expression Strains

To investigate the function of FSE1, the nucleotide of the gene was knocked out via the homologous recombination strategy (Figure 1A). Two rounds of PCR diagnosis and subsequent confirmed the correct integration of the recombinant fragments into the *FSE1* locus and successful knockout of the gene from the genome (Figure 1B). After single conidia isolation and verification for the non-presence of *FSE1* nucleotide, three independent mutants were generated, named ∆*FSE1*, and selected for the following analysis. Since the three mutants showed similar phenotypes in vegetative growth, conidiation, and pathogenicity, only one was selected for the construction of the OE mutant (Figure 1C). The PCR and Western blotting analyses confirmed the expression of the fusion protein FSE1-FLAG in the OE mutant, which was named ∆*FSE1*/*FSE1*-OE (Figure 1D,E).

### 3.3. FSE1 Is Not Required for Vegetative Growth and Conidiation

**Figure 2:**
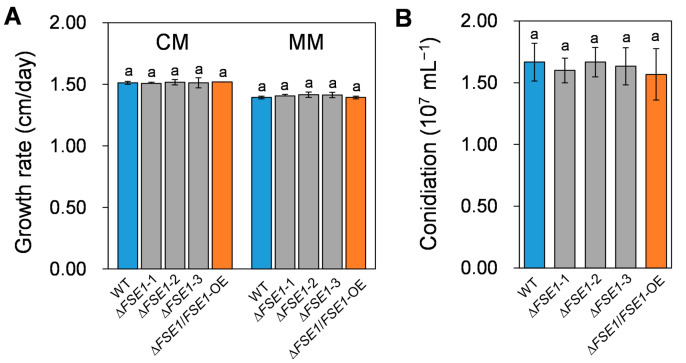
Growth rate and conidiation assays of the *FSE1* knockout and overexpression mutants. (**A**) Foc TR4 strains were grown on complete (CM) or minimal agar medium (MM) for 5 d, after which the colony growth rates were calculated. (**B**) Foc TR4 strains were grown in liquid complete medium for 3 d, and the conidiation production was counted with Hemocytometer. Bars represent standard deviations (SD). Data are shown as the means ± SD, and columns with different letters indicate significant difference (*p* < 0.05).

To investigate the roles of FSE1 in growth and conidiation, the mutant strains were cultured on/in different media, and their phenotypes were accessed. As shown in Figure 2A, the ∆*FSE1* and ∆*FSE1*/*FSE1*-OE mutants showed similar colony growth rates compared to the wild type (WT), which were about 1.5 and 1.4 cm/day on complete medium and minimal medium, respectively. In addition, the mutant strains produced the same amount of conidia as WT when cultured in a liquid complete medium (Figure 2B). These results suggested that FSE1 is not required for normal vegetative growth or conidiation in Foc TR4.

### 3.4. FSE1 Is Involved in the Pathogenicity of Foc TR4

**Figure 3:**
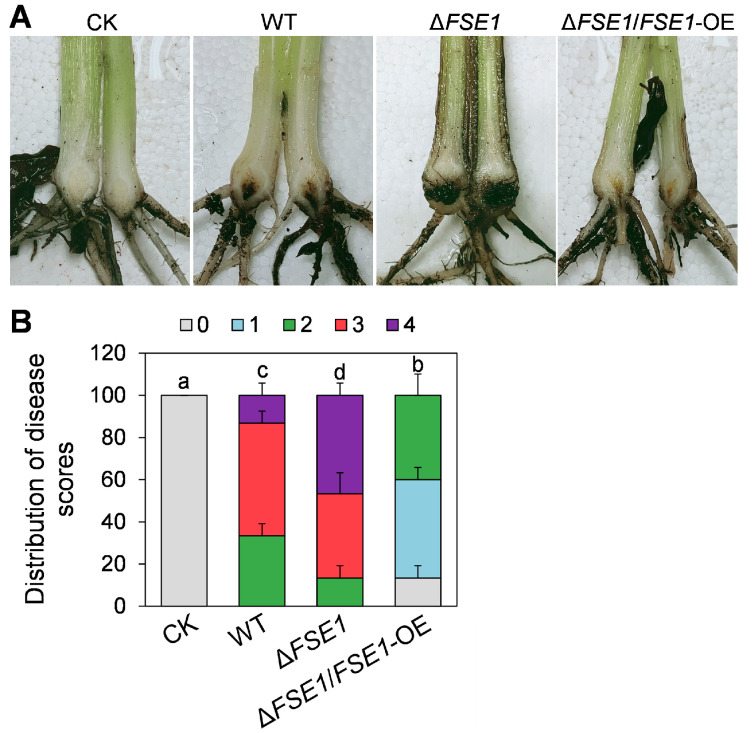
Pathogenicity assays of the *FSE1* knockout and overexpression mutants. Banana plantlets were inoculated with conidia suspension of Foc TR4 strains; after incubation for 5 weeks, the banana pseudostem were sampled and used for the disease symptoms and disease scores investigation. (**A**) Disease symptoms of rhizome and pseudostem of banana plantlets after infection for 5 weeks. (**B**) Distribution of disease scores. Treatments with different letters indicate significant difference (*p* < 0.05). CK, banana plantlets treated with ddH_2_O.

To determine the roles of FSE1 in pathogenicity, the conidia suspension of the mutants was inoculated into banana roots. At 5 weeks post-inoculation, the pseudostem browning was measured for disease scores (Figure 3A). The results (Figure 3B) revealed that 33% of the plantlets treated with WT had a disease score of 2, 53% had a score of 3, and 13% had a score of 4. In comparison, only 13% of those plantlets treated with ∆*FSE1* had a score of 2, 40% had a score of 3, and 47% had a score of 4, indicating a significant increase in disease index. Meanwhile, the ∆*FSE1*/*FSE1*-OE mutant showed a decreased disease index compared with WT, with no plantlets at disease scores above 3 and 4.

### 3.5. FSE1 Is Distributed in Vesicles of Foc TR4 and Localized in Cytoplasm and Nuclei of N. benthamiana Cells

**Figure 4:**
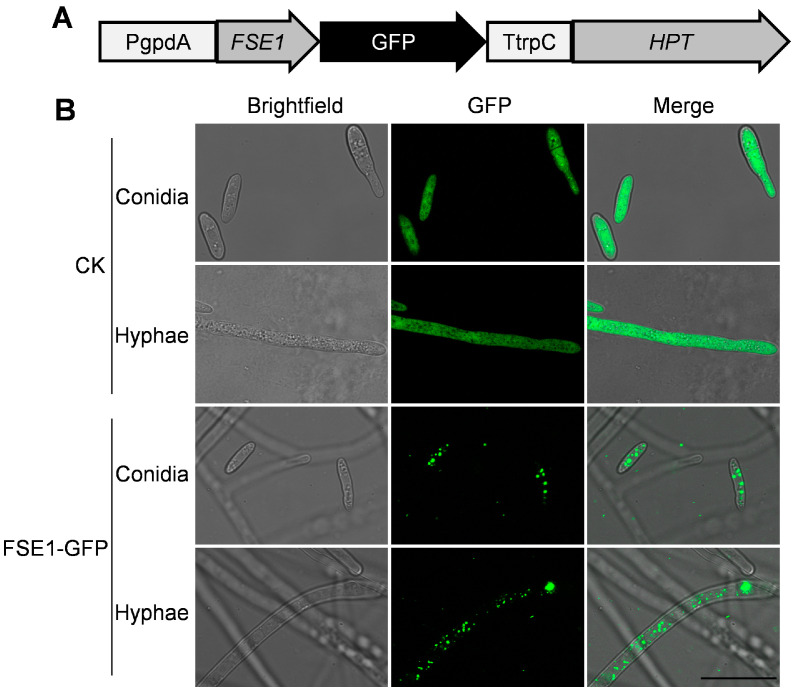
FSE1-GFP is distributed in vesicles of Foc TR4. (**A**) Expression cassette of FSE1-GFP. (**B**) Fluorescence microscopes of conidia and hyphae of Foc strains. Scale Bar = 20 μm.

To analyze the subcellular localization of FSE1 in Foc TR4, the transformants expressing FSE1-GFP fusion protein were constructed (Figure 4A). The microscope observation revealed punctiform fluorescence of FSE1-GFP in the cytoplasm of both conidia and hyphae. In comparison, the CK expressing GFP alone showed strong fluorescence throughout the cell (Figure 4B).

**Figure 5:**
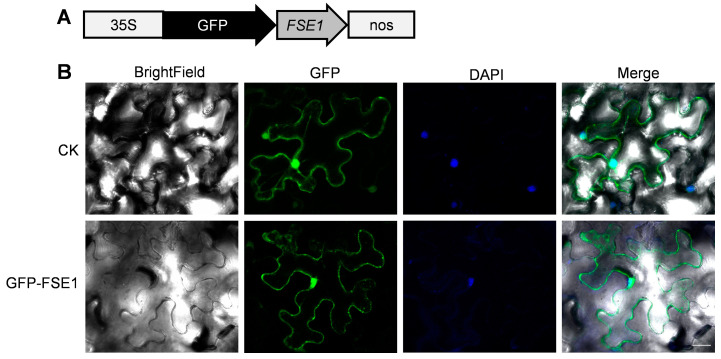
FSE1 is distributed in cytoplasm and nuclei of *N. benthamiana* epidermal cells. (**A**) Expression cassette of GFP-FSE1. (**B**) Fluorescence microscopes. CK, cells expressing empty GFP; DAPI, 4′,6-diamidino-2-phenylindole. Scale Bar = 25 μm.

To further analyze the subcellular localization of FSE1 in plant cells, the GFP-FSE1 fusion protein was transiently expressed in leaves of *N. benthamiana* by agroinfiltration. Microscopic analysis revealed that the fluorescence of GFP-FSE1 was distributed in both cytoplasm and nuclei of *N. benthamiana* cells (Figure 5).

### 3.6. FSE1 Interacted with MYB Family Transcription Factor EFM

**Figure 6:**
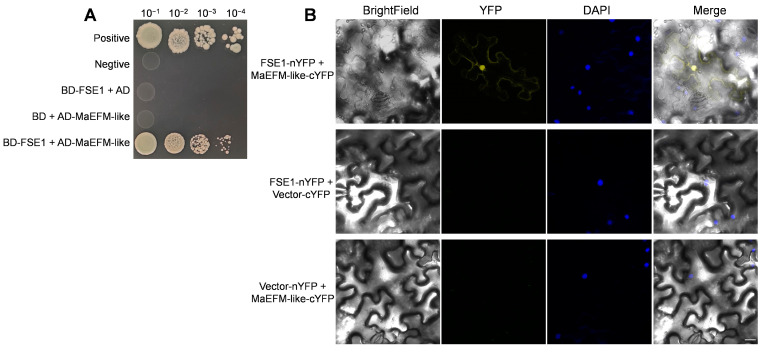
Verifying interaction between FSE1 and MaEFM-like. (**A**) Yeast two-hybrid (Y2H) assays showing FSE1 interact with MaEFM-like. Yeast cell were grown on SD/-Trp-Leu-His-Ade plates containing 125 ng mL^−1^ aureobasidin A (ABA). (**B**) BiFC analysis of *in planta* interaction between FSE1 and MaEFM-like in *N. benthamiana* epidermal cells. DAPI, 4′,6-diamidino-2-phenylindole. Scale Bar = 25 μm.

To identify potential targets of FSE1 in banana cells, the coding sequence without SP was introduced into pGBKT7 as the bait, and the yeast two-hybrid assay was performed by screening a banana root cDNA library. After the initial screening, a predicted protein (accession XM_009390653.2) was identified as a potential interacting protein of FSE1. Phylogenetic tree analysis showed that the predicted protein was homologous to the MYB family transcription factor EFM from *Arabidopsis* (Figure S4A), and therefore, the predicted protein was named MaEFM-like. The *MaEFM*-like gene was amplified by RT-PCR and verified by sequencing. The result showed that the full-length cDNA of MaEFM-like is 1098 bp, encoding 366 amino acids, and contains two MYB DNA-binding domains. The BLASTP search in the NCBI database and the phylogenetic tree showed that MaEFM-like protein was conserved in *Musa* species (Figure S4B). Then the full-length cDNA of *MaEFM*-like was introduced into pGADT7 vector and co-expressed with pGBKT7-*FSE1*. The verification assay showed that FSE1 strongly interacted with MaEFM-like (Figure 6A).

Furthermore, a BiFC assay was conducted in *N. benthamiana* leaves to confirm the *in planta* interaction between FSE1 and MaEFM-like. The FSE1-nYFP and MaEFM-like-cYFP constructs were introduced into *A. tumefaciens* and co-infiltrated into *N. benthamiana* leaves. Leaves from plants infiltrated with either of the fusion proteins alone or in combination with the empty vector showed no fluorescence (Figure 6B); in comparison, strong YFP fluorescence could be observed when the two proteins were co-expressed. Moreover, the YFP fluorescence was co-localized with DAPI fluorescence, indicating that FSE1 interacted with MaEFM-like in the nuclei of *N. benthamiana* cells. The above results strongly suggested that FSE1 physically interacts with MaEFM-like.

### 3.7. FSE1 Supressed the MaEFM-Like-Induced Cell Death

**Figure 7:**
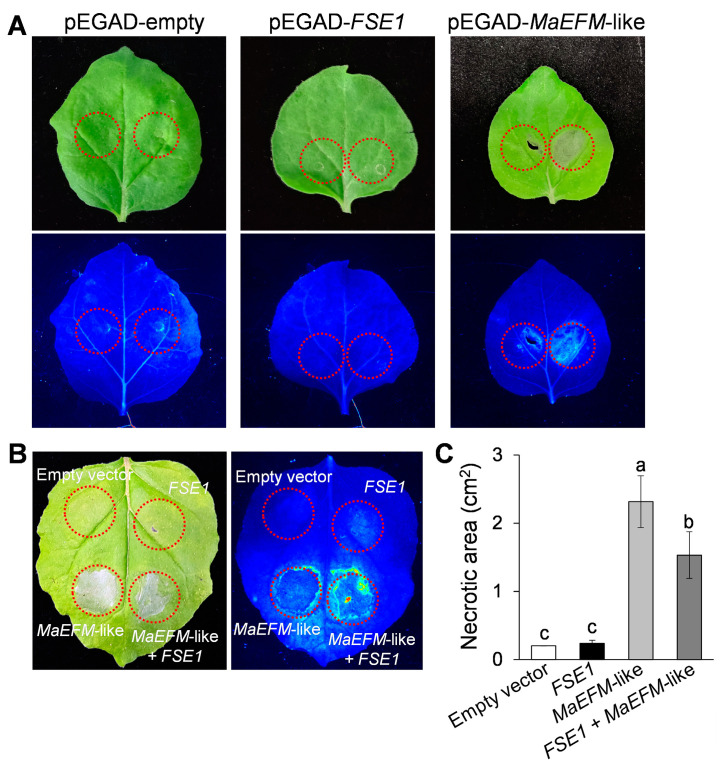
MaEFM-like induced cell death in tobacco leaves and FSE1 suppressed the cell death induced by MaEFM-like. (**A**) Leaves expressing empty vector, *FSE1*, or *MaEFM*-like were photographed under normal light (**up**) and UV illumination (**bottom**). (**B**) FSE1 suppressed the cell death induced by MaEFM-like. (**C**) Statistical analysis of necrotic area in tobacco leaves. Data are shown as the means ± SD, and columns with different letters indicate significant difference (*p* < 0.05).

To investigate the function of MaEFM-like in plant disease response, the protein was overexpressed in *N. benthamiana* leaves by infiltrating with A. tumefaciens harboring the pEGAD-MaEFM-like plasmid. Two days post-infiltration, significant necrosis was observed in the area expressing MaEFM-like, while no necrosis was observed on the leaves infiltrated with either the empty pEGAD or pEGAD-FSE1 plasmids (Figure 7A). Additionally, the necrotic area caused by co-expression of MaEFM-like with FSE1 was significantly smaller than that caused by MaEFM alone (Figure 7B,C). The result suggested that MaEFM-like could induce cell death in plant cells, and FSE1 can suppress the MaEFM-like-induced cell death.

## 4. Discussion

Identification and functional analysis of fungal effectors can provide insights into key processes of fungi-host interaction. Over the past decades, an increasing number of fungal effectors have been identified and well-investigated in many phytopathogenic fungi. However, for the banana wilt disease causal agent Foc TR4, only a few effectors have been experimentally characterized [16,17,22]. Based on effector prediction procedures via secretome analysis and the machine-learning tool EffectorP [18,23], an effector candidate coding gene *FSE1* was identified in the genome of Foc TR4. Although no conserved domains were identified in FSE1, the protein contains 16 cysteines (6.25% of total amino acids) and a 19 aa N-terminal signal peptide (Figure S1), which matches the sequence characteristics of effectors [24]. Through BLAST searching against the Top 10 fungal pathogens [2] and other *Fusarium* species, it was found that FSE1 and its homologs proteins are only conserved in in some *Fusarium* species, especially *F. oxysporum formae* speciales (Figure S2). In phytopathogenic fungi, the presence of effectors is closely associated with the determination of host range, for example, *Fusarium* and *Alternaria* species [12,25]. Taken together, we suggest that FSE1 is the *Fusarium* special effector involved in their pathogenicity.

To investigate the function of FSE1, its nucleotide was deleted from the genome of FocTR4 and over-expressed based on the knock-out mutant. The in vitro assays revealed that *FSE1* mutants showed comparable colony growth rates and conidia production (Figure 2), indicating that FSE1 is not required for vegetative growth and conidiation. Fungal effectors are divided into apoplastic and cytoplasmic effectors [7], with apoplastic effectors primarily functioning in the apoplast or binding to the fungal cell wall to shield fungus from reception by plant immunity [26,27], while cytoplasmic effectors are delivered into the plant cell to exert their functions and do not influence fungal growth [28]. Considering this, we deduce that FSE1 was probably a cytoplasmic effector. After inoculation to banana plantlets, all the WT and the *FSE1* mutant strains infected the host and provoked disease symptoms, suggesting that FSE1 is not required for the initial infection process of Foc TR4 to banana root. Moreover, the disease index assay revealed that ∆*FSE1* increased pseudostem browning and plant wilt in comparison with WT, while the ∆*FSE1*/*FSE1*-OE mutant showed a decreased disease index (Figure 3 and Figure S5). *F. oxysporum* is considered a hemibiotrophic pathogen because it begins its infection cycle as a biotrophy but later changes to a necrotrophy [29,30]. Therefore, we deduced that FSE1 plays an important role in maintaining biotrophy and colonization in the vascular systems of banana plants.

Determining the sub-cellular localization of effectors can improve the understanding of their function in the interaction with the host. In the Foc TR4 mutant expressing FSE1-GFP, the fusion protein was localized in the cytoplasm with punctiform expression (Figure 4). Although effector delivery systems are well characterized in bacteria, oomycetes, and nematodes [9,10,31,32], effector delivery mechanisms in fungi remain elusive. Some studies have suggested that effector secretion in filamentous fungi involves the trafficking of secretory vesicles to a central organizing center called Spk [33,34]. It is therefore possible that FSE1 is secreted via vesicle trafficking in Foc TR4. Fungal effectors are known to target various subcellular compartments of host plants to overcome physical barriers, inhibit immune perception, and manipulate plant physiology for nutrients [35]. Our findings show that FSE1 is distributed in both cytoplasm and nuclei of *N. benthamiana* cells (Figure 5). Recent evidence suggests that nucleus-targeted effectors can manipulate host transcriptional machinery to interfere with plant immunity during plant-pathogen interactions [7,28]. Consequently, we searched for the target protein of FSE1 in banana cells, and the Y2H and BiFC results showed that FSE1 physically interacted with an MYB transcription factor, MaEFM-like (Figure 6).

Plant TFs play important roles in defense responses to pathogens [36,37,38]. Many studies have documented that fungal effectors directly target plant TFs to exert their functions [39,40,41]. In this study, overexpression of MaEFM-like induced typical necrosis and cell death in *N. benthamiana* leaves (Figure 7A), which was in accordance with the previous report that some MYB TFs are activators of the hypersensitive reaction (HR) in response to pathogen attack [42,43]. Moreover, FSE1 suppressed this HR when co-expression with MaEFM-like. Hemibiotrophic pathogens have to maintain biotrophy colonization before entering the necrotrophic stage. During the biotrophy stage, the pathogens could secrete effectors to inhibit host defenses and suppress cell death. For example, the *Magnaporthe oryzae* effector AvrPiz-t interacts with the bZIP-type transcription factor APIP5 in the cytoplasm and suppresses its transcriptional activity at the necrotrophic stage [40]; the *Colletotrichum gloeosporioides* effector CgNLP1 disrupts nuclear localization of necrosis-induced TF HbMYB8-Like to suppress plant HR [41]. These results strongly suggested that the pathogenicity of FSE1 was achieved through suppression of the MaEFM-mediated HR.

In summary, our study has identified a novel effector, FSE1, which plays a crucial role in the pathogenicity of Foc TR4. Our results have demonstrated that FSE1 targets the MYB transcription factor MaEFM-like to maintain biotrophy in banana plants. This discovery enhances our understanding of the mechanisms employed by Foc TR4 to evade host defenses and cause disease.

## Data Availability

Not applicable.

## Supplementary Materials

The following supporting information can be downloaded at: https://www.mdpi.com/article/10.3390/jof9040472/s1, Figure S1: Nucleotide sequence and deduced amino acid sequence of FSE1. Shading indicates the amino acid sequences of the signal peptide and cysteine residues; Figure S2: Phylogenetic tree of FSE1 with homologs proteins from other fungi; Figure S3: Quantitative real-time PCR analysis of the transcription of *FSE1* after inoculation to banana plantlets for 3, 5, and 7 d; Figure S4: Phylogenetic trees of MaEFM-like with homologs proteins from *Arabidopsis thaliana* and *Musa* species; Figure S5: Disease symptoms of banana plantlets after inoculation for 5 weeks; Table S1: Primers used in this study.
