# Supplementary material for: A Novel Effector, FSE1, Regulates the Pathogenicity of Fusarium oxysporum f. sp. cubense Tropical Race 4 to Banana by Targeting the MYB Transcription Factor MaEFM-Like
Source: J Fungi (Basel). 2023 Apr 14;9(4):472. doi: 10.3390/jof9040472 (PMC10144757; doi:10.3390/jof9040472)
Supplement: Supplementary file 1 [file jof-09-00472-s001.zip › jof-2243812-supplementary.pdf]

1 ATGCGGTTCTCAGCTGCGTTCCTCGCAACCGTCCTCGGTGCCGTTAATATCAATGCGCAA

1 M R F S A A F L A T V L G A V N I N A Q

61 TGCATCGACGGCCATCGAGAGGTTATCAGCCCCGGCTACACCGTTGAGTACAAGTGCAAC

21 C I D G H R E V I S P G Y T V E Y K C N

121 TTCGTCCGACTGGGCGAGACTCACAATGGCGTTCTCTCTGAAAAGGCCTGCGCTGAGATG

41 F V R L G E T H N G V L S E K A C A E M

181 TGTCGCGATGCTGGTAGCTCTGTCTGTACTTATCATCCGCCCACCAAACGCTGTGTCGTC

61 C R D A G S S V C T Y H P P T K R C V V

241 GGCAAGGATGGTGGCAAGGAAATGGCCAGTAACGGCGCTATATACATGATGAAAGTCGAC

81 G K D G G K E M A S N G A I Y M M K V D

301 GAGCCTGAGATTGAGGACCCTTTTGCTGAAGATGAAGACCCTTTTTCTGTTGATTGCGAA

101 E P E I E D P F A E D E D P F S V D C E

361 GCTGAGAAGCAGGCTTGTGAGAGTGGTCAAAAGGCTTGTCTGGAGCGGGAGAAGAAGCTC

121 A E K Q A C E S G Q K A C L E R E K K L

421 AAGGCCTCTCATGCTGGTTTAGAGGCCAAGAACAATAGTCTTGAAGCACGCATCAAGAAT

141 K A S H A G L E A K N N S L E A R I K N

481 ATCATGCAATCAAATTGCCCTAGTCAGCATGGTAAGTTTGGGGTTGTTAACAACCGAGAG

161 I M Q S N C P S Q H G K F G V V N N R E

541 TACCGCTTCTGGTGCGGCCGACACCACAGCCCCGAAGGATTCAAAGAAGAGCTTCCCGAG

181 Y R F W C G R H H S P E G F K E E L P E

601 ATTTACACCATGGCAGACTGCGTCGATCAGTGTAGTCGCAAGGCCTGGTGCAATCATGTC

201 I Y T M A D C V D Q C S R K A W C N H V

661 TTACACGGCATTCACAAAAACAAGTGCCGTTTGTTCGAGAGTCCCAAGGTTTCCGCAGCC

221 L H G I H K N K C R L F E S P K V S A A

721 ACAATGCCAGGTCTTGCCACTGGTGACTGGAACTGTGGTGTCAAGAAGTAG

241 T M P G L A T G D W N C G V K K *

Figure S1. Nucleotide sequence and deduced amino acid sequence of FSE1. Shading indicates the amino acid sequences of the signal peptide and cysteine residues.

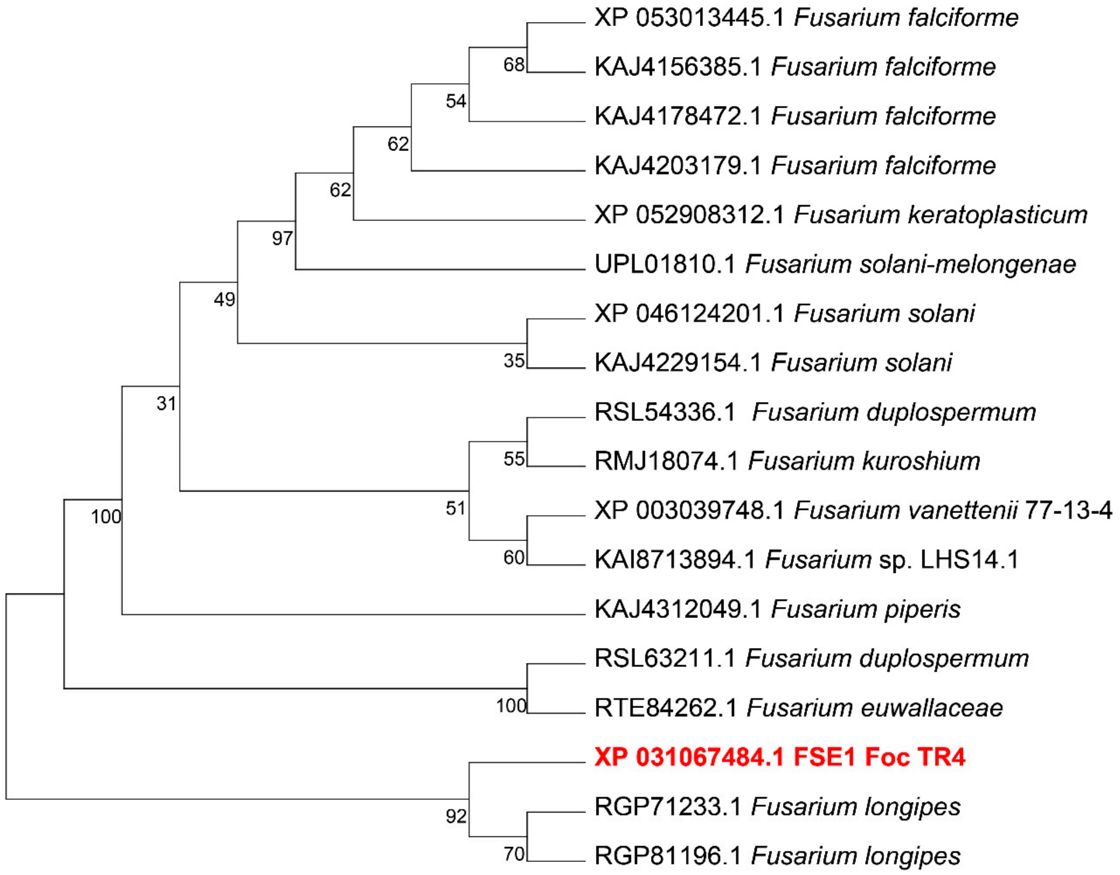

Figure S2. Phylogenetic tree of FSE1 with homologs proteins from several *Fusarium* species.

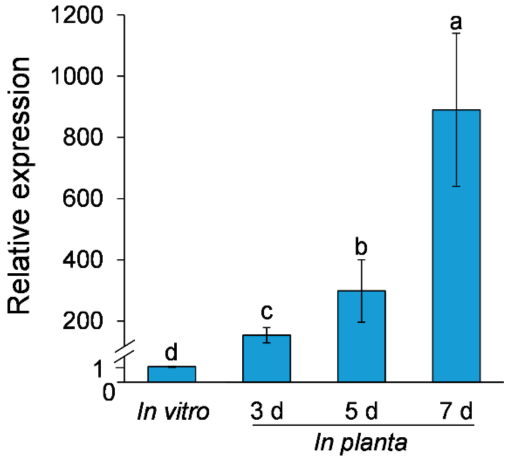

Figure S3. Quantitative real-time PCR analysis of the transcription of *FSE1* after inoculation to banana plantlets for 3, 5, and 7 d. The relative expression level is assessed based on $2^{-\Delta\Delta Ct}$ method through normalization against the transcription level of the *In vitro* sample. *Actin* has been used as the internal control. Bars represent standard deviations (SD). Columns with different letters indicate significant difference ($P < 0.05$).

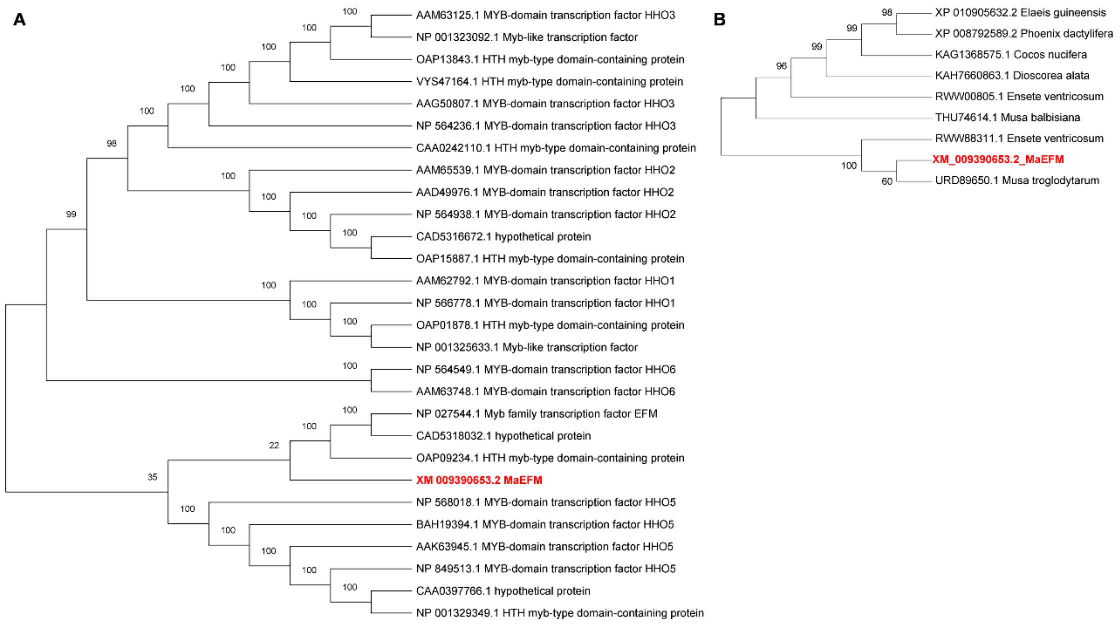

Figure S4. Phylogenetic trees of MaEFM-like with homologs proteins from *Arabidopsis thaliana* and *Musa* species.

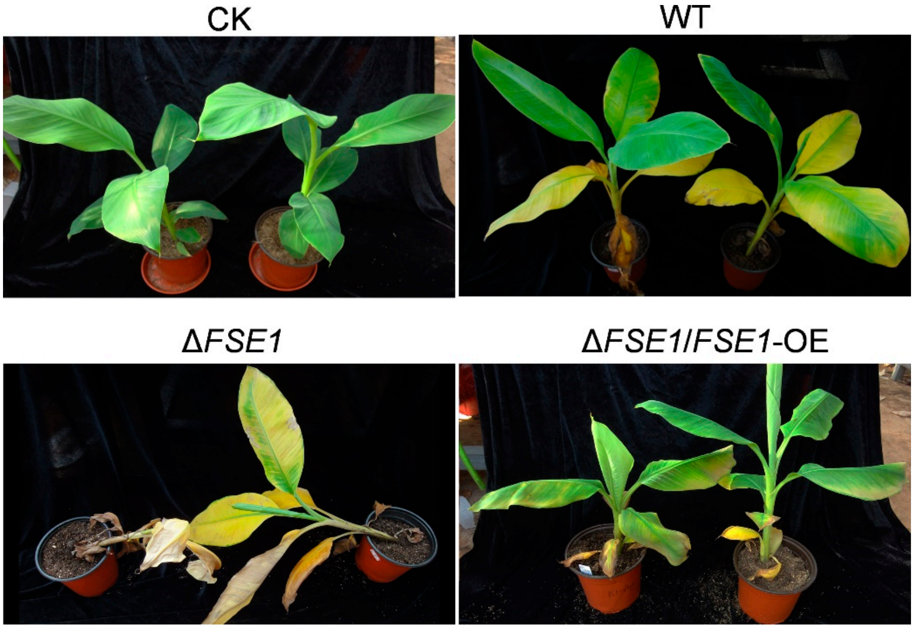

Figure S5. Disease symptoms of banana plantlets after inoculation for 5 weeks.
